# Chemometric Model for Rapid Determination of Syringyl/Guaiacyl Ratio in Non‐Wood by FT‐NIR Spectroscopic Data

**DOI:** 10.1002/ansa.70005

**Published:** 2025-03-11

**Authors:** M. Nashir Uddin, Taslima Ferdous, Yangcan Jin, M. Mostafizur Rahman, M. Sarwar Jahan

**Affiliations:** ^1^ BCSIR Laboratories Bangladesh Council of Scientific and Industrial Research (BCSIR) Dhaka Bangladesh; ^2^ Department of Applied Chemistry and Chemical Engineering University of Dhaka Dhaka Bangladesh; ^3^ Jiangsu Co‐Innovation Center of Efficient Processing and Utilization of Forest Resources Jiangsu Key Lab of Pulp and Paper Science and Technology Nanjing Forestry University Nanjing China

**Keywords:** chemometric modelling, multispecies of non‐wood, syringyl/guaiacyl ratio

## Abstract

The present study is to develop a cost‐effective, non‐destructive and rapid method for quantification of syringyl/guaiacyl (S/G) ratio content in non‐wood lignin, which is based on FT‐NIR spectroscopic data and chemometric modelling techniques. The S/G ratio in 22 non‐wood lignins was determined by wet chemical method. Then the same samples were run with FT‐NIR, and the spectroscopic data were pre‐processed with Savitzky–Golay (S–G) on their 1st and 2nd derivatives. As chemometric models, principal component regression (PCR) and partial least square regression (PLSR) were assessed for quantification of S/G ratio in non‐wood lignin with raw and pre‐treated FT‐NIR spectral data. Finally, for quantification of S/G ratio, PLSR showed the best predictive results (*R* ^2^ = 99.90%) with FT‐NIR data after treating them with S–G filtered with its derivatives and leverage correction. This rapid and cost‐effective method is being proposed for the determination of S/G ratio in non‐wood lignin.

## 1 Introduction

Lignin is a complex natural polymer made up of *p*‐hydroxyphenyl (H), guaiacyl (G) and syringyl (S) units. These units are connected by ether and carbon–carbon bonds in an irregular, repeating pattern [1]. Syringyl (S) and guaiacyl (G) content lignin and its ratio (S/G) are important characteristics for delignification selectivity of lignocellulosic biomass. These characteristics depend on factors like the origin of the tree, growth conditions, climate, species and so on [2, 3, 4, 5, 6]. The higher S/G ratio in lignocelluloses is easier to delignify in chemical pulping [7, 8].

Traditionally, the S/G ratio is determined by chemical degradation of lignin followed by chromatography analysis through thioacidolysis, permanganate oxidation and alkaline oxidation with nitrobenzene [9, 10, 11, 12, 13]. The pyrolysis–gas chromatography–mass spectrometry (Py–GC/MS) was also used for syringyl/guaiacyl (S/G) ratio determination [14, 15, 16, 17, 18]. These methods of determining S/G ratio requires chemicals, which create waste, and the process is tedious and time consuming to complete the testing procedure for every sample.

Several researchers have investigated the S/G ratio of non‐wood lignins. del Río et al. [19] used pyrolysis–gas chromatography–mass spectrometry (Py–GC/MS) to analyse lignins from hemp (Cannabis sativa), flax (Linum usitatissimum), jute (Corchorus capsularis), sisal (Agave sisalana) and abaca (Musa textilis). They found that hemp and flax lignins were predominantly composed of guaiacyl units, while jute, sisal and abaca lignins were primarily composed of syringyl units. Nakagawa‐Izumi et al. [20] also employed Py–GC/MS to investigate the lignin in the thermomechanical pulp of oil palm empty fruit bunch. Their Py–GC/MS derived S/G molar ratios correlated well with the syringaldehyde/vanillin (Sa/Va) molar ratios obtained by alkaline nitrobenzene oxidation (ANO) for the raw empty fruit bunch material. More recently, Rosado et al. [21] studied rice straw and rice husk lignin using Py–GC/MS and 2D NMR. They observed that these lignins were rich in guaiacyl units and depleted in p‐hydroxyphenyl and syringyl units, with H:G:S compositions of 7:81:12 for rice husks and 5:71:24 for rice straw.

ANO has been used to investigate the structural composition of lignin from various sources. Ferdous et al. [22] analysed different parts of the banana plant, finding that p‐hydroxybenzaldehyde was the main product in banana stem (10.8%) and leaf (8.89%) lignins. In contrast, the primary degradation products in banana peduncle lignin were syringaldehyde (11.4%) and vanillin (8.27%). ANO was also used by Ferdous et al. [23] to determine the S/G ratios of bagasse (0.918) and corn stalk (1.142) lignins.

However, application of multivariate analysis assisted chemometric modelling techniques with spectroscopic data as an alternative approach enables rapid and dependable analysis using non‐destructive methods. In this technique, at first, desired parameter is quantified with a number of samples with some chemical or other traditional methods, then spectroscopic data are collected by running the same samples in any spectroscopic instrument. The data from traditional methods and the spectroscopic instrument are modelled with some chemometric calibration techniques. The best performing models are then used further only by using the spectroscopic data of any new sample (s) for quantifying that particular parameter. Once the method is developed, no chemical is needed for further analysis; therefore, this method is less costly and requires comparatively less time than traditional methods [24, 25, 26, 27, 28]. An additional advantage of this method is that it typically requires no sample preparation before analysis. Chemometric modelling based on multivariate analysis of spectroscopic data is a comparatively new technique for developing an analytical method for quantification of lignocellulosic parameters in pulp and lignin. Recently, such methods have been studied for the determination of chemical composition of wood, wood properties, pulp properties and so on [11, 29, 30, 31, 32, 33]. Similar methods have also been developed for prediction of paper properties like tensile index and brightness [34, 35].

Most recently, our research group has developed a multivariate analysis assisted chemometric models based on spectroscopic data from NIR, Fourier transform near‐infrared (FT‐NIR), FT‐Raman and similar other instruments for determining non‐wood pulp properties [33, 36, 37]. Chemometric models for quantification of chemical components such as, lignin, pentosan, holocellulose and α‐cellulose in non‐wood pulp have been developed with artificial neural network (ANN) calibration techniques (*R* ^2 ^≈ 0.99) by using FT‐NIR data [38]. Also, similar models for determination of α‐cellulose and R_10_ with UV and pentosan, R_18_, viscosity and brightness in dissolving pulp from jute cutting and jute caddis with FT‐NIT data and partial least square regression (PLSR) model, was reported [32, 33]. For α‐cellulose quantification, the PLSR model performed the best, with a coefficient of determination (*R* ^2^) of 0.94%, when the data were pre‐treated with mean normalisation [33]. To describe the brightness response of Eucalyptus mechanical pulp under different bleaching conditions, a radial basis function neural network (RBFNN) predictive model was built with NIR spectroscopic data [39].

For determination of S/G ratio in lignin from different wood sources, chemometric models have been developed with multivariate calibration of near infrared spectral data [24, 25, 26, 28, 40], FT‐Raman spectroscopy [15, 41, 42], Raman spectroscopy [43] and NMR [44, 45] for lignin from different wood species like *Eucalyptus globulus*, *Pinus radiata*, Norway spruce and so on.

No research has been reported for quantification of S/G ratio in non‐wood samples of multiple species with multivariate analysis assisted chemometric model‐based method so far. Non‐wood samples are anatomically different from wood species. Chemical compositions are also different. FT‐NIR spectroscopy is one of the most common tools to characterise lignin. It is also easy and rapid to operate. This study of developing chemometric model with FT‐NIR spectroscopic data would provide a new cost‐effective, non‐destructive and rapid method for quantifying the S/G ratio in lignin from multiple species non‐wood samples. The method utilises chemometric modelling of FT‐NIR spectroscopic data from 22 different non‐wood agricultural residue samples.

## 2 Materials and Methods

### 2.1 Samples

A total of 22 non‐wood samples, mostly from crop residue, were collected from different parts of the country which are listed in Appendix 1. These crop residues were milled to 40–60 mesh size and extracted with ethanol:toluene (2:1) using a Soxhlet extractor (T204 om88). Klason lignin content was then determined (T222 om83). Before ANO, moisture content in all samples were determined using Tappi standard method T 264.

### 2.2 Quantification of S/G Ratio

The S/G ratio in different non‐wood lignins were determined by ANO method [23].

### 2.3 ANO

First, a 10 mg sample of extractive‐free milled non‐wood material was placed in a bomb, and 4 mL of 2 M NaOH along with 0.25 mL of nitrobenzene were added. The mixture was heated at 170°C for 2 h. Following this, a 0.1 M NaOH internal standard solution containing 3‐ethoxy‐4‐hydroxybenzaldehyde (0.2–0.4 g/L, 1 mL) was introduced. The reaction mixture was extracted three times with 15 mL of dichloromethane. The aqueous phase was then acidified to pH ∼ 1 using 4 M HCl and extracted twice with 20 mL of dichloromethane and once with 15 mL of ethyl ether. The combined organic phases were washed with 20 mL of water, dried over Na2SO4 and then filtered to remove insoluble inorganic materials. The solution was evaporated to dryness and silylated with BSA at 105°C for 5 min. The silylated compounds were analysed using gas chromatography with a Shimadzu 17 A GC‐FID under the specified conditions.

Injection volume: 1μ1
Gas Column: NB‐l (GL science) fused‐silica capillary column (length, 30m; 0.25 mm i.d.), Column temperature: 150°C, 15 min −3°C/min•180°C −10°C/min•280°C
Injection temperature: 250°C
Detector temperature: 280°C
Column flow rate of He gas: 1.9 mL/min
Splitting ratio: 60:1

### 2.4 FT‐NIR Spectroscopic Data Acquisition

FT‐NIR spectroscopy was conducted using a PerkinElmer FT‐NIR spectrometer (Model Frontier, PerkinElmer, USA) equipped with an indium gallium arsenide (InGaAs) detector. The spectral range covered was 10,000–4000 cm^−^¹. For each sample, 32 scans were collected at a spectral resolution of 16 cm^−^¹ with an interval of 4 cm^−^¹. The scans were averaged and recorded as reflectance percentage (%R). Data processing was performed using PerkinElmer Spectrum (Version 10.4.4) software.

In the spectra, each wavenumber corresponds to a reflectance value, which is critical for spectral analysis. Given the vast number of reflectance values at different wavenumber, these data points (spectroscopic variables) are numerous and interrelated, necessitating dimensionality reduction and pre‐processing to manage the complexity.

### 2.5 Pre‐Processing of Spectroscopic Data

After acquisition of spectroscopic data, they were pre‐processed with different de‐noising techniques commonly used in chemometric techniques [46].

The data were pre‐treated with (1) S–G filtering with 1st derivatives, (2) S–G filtering with 2nd derivatives, (3) S–G filtering with 1st and 2nd derivatives simultaneously and (4) leverage correction.

### 2.6 Chemometric Model Development and Validation

Two widely used chemometric calibration techniques, principal component regression (PCR) and PLSR, were evaluated for quantification of S/G ratio of lignin from non‐wood using FT‐NIR spectroscopic data [47]. The most effective technique was selected for method development.

To validate the developed models for quantifying the S/G ratio, a sixfold cross‐validation (CV) dataset was created for each model [48]. CV is commonly used for applying in machine learning to compare and select a model for a given predictive modelling problem because it is easy to understand, easy to implement and results in skill estimates that generally have a lower bias than other methods.

### 2.7 Leverage Correction

In regression analysis, leverage measures how far the independent variable values of a particular observation are from those of the other observations. Essentially, leverage quantifies the influence or importance of a sample, object or variable on the calibration model. A leverage value close to zero signifies that the sample or variable has minimal impact on the calibration model [49].

Leverage correction utilises leverage values to estimate prediction errors without performing actual predictions. This correction is achieved by adjusting the residuals of the dependent variable (*y*) based on the sample leverages (*h_i_*). This approach helps to account for the influence of individual observations on the overall model, improving the accuracy of the error estimates:

$$\;f_{ij}^{\mathrm{corrected}}=\frac{f_{ij}}{1-h_{i}}$$

Thus, the higher the leverage *h_i_*, the larger 1/(1 − *h_i_*), and extreme samples will have larger prediction residuals than average ones. This is an effective way to take into account the influence of these samples may have on the model [50].

## 3 Results and Discussion

The S/G ratios in 22 non‐wood pulp were analysed by ANO (Table 1). As shown, it varied from 0.70 in banana stem to 3.10 in jute fibre.

**TABLE 1:** Syringyl/guaiacyl (S/G) ratio in non‐wood agricultural residues.

Sl No.	Sample Name	S/G Ratio	Sl No.	Sample Name	S/G Ratio
1	Bagasse	0.92	12	Jute fibre	3.10
2	Bamboo	1.02	13	Jute stick	1.28
3	Banana pseudo stem	0.70	14	Kash stalk	2.51
4	Banana leaf	1.12	15	Kaun straw	2.26
5	Banana peduncle	1.38	16	Mustard stalk	1.21
6	Cassava stalk	1.55	17	Mulberry stalk	1.14
7	Chia stalk	1.41	18	Okra stalk	1.05
8	Corn stalk	1.23	19	Pineapple leaf	1.66
9	Cotton stalk	1.14	20	Red lentil stalk	2.56
10	Dhaincha stalk	1.49	21	Rice straw	2.09
11	Eggplant stalk	1.63	22	Wheat straw	2.05

**FIGURE 1:**
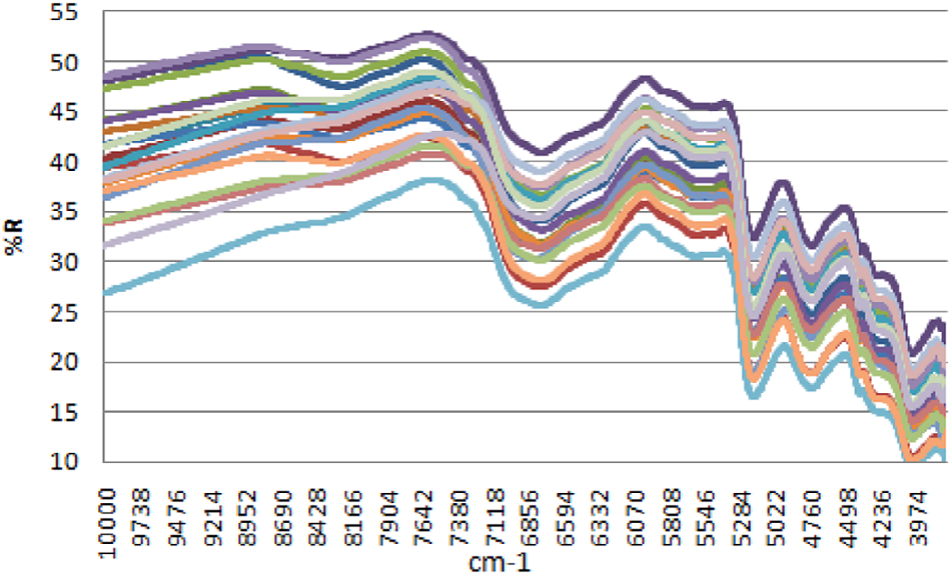
FT‐NIR spectroscopic data of reflectance (%R) against wavenumber (cm^−1^).

FT‐NIR spectroscopic data of reflectance (%R) against wavenumber (cm^−1^) have been depicted in Figure 1 ranging from 10,000 to 4000 cm^−1^.

**FIGURE 2:**
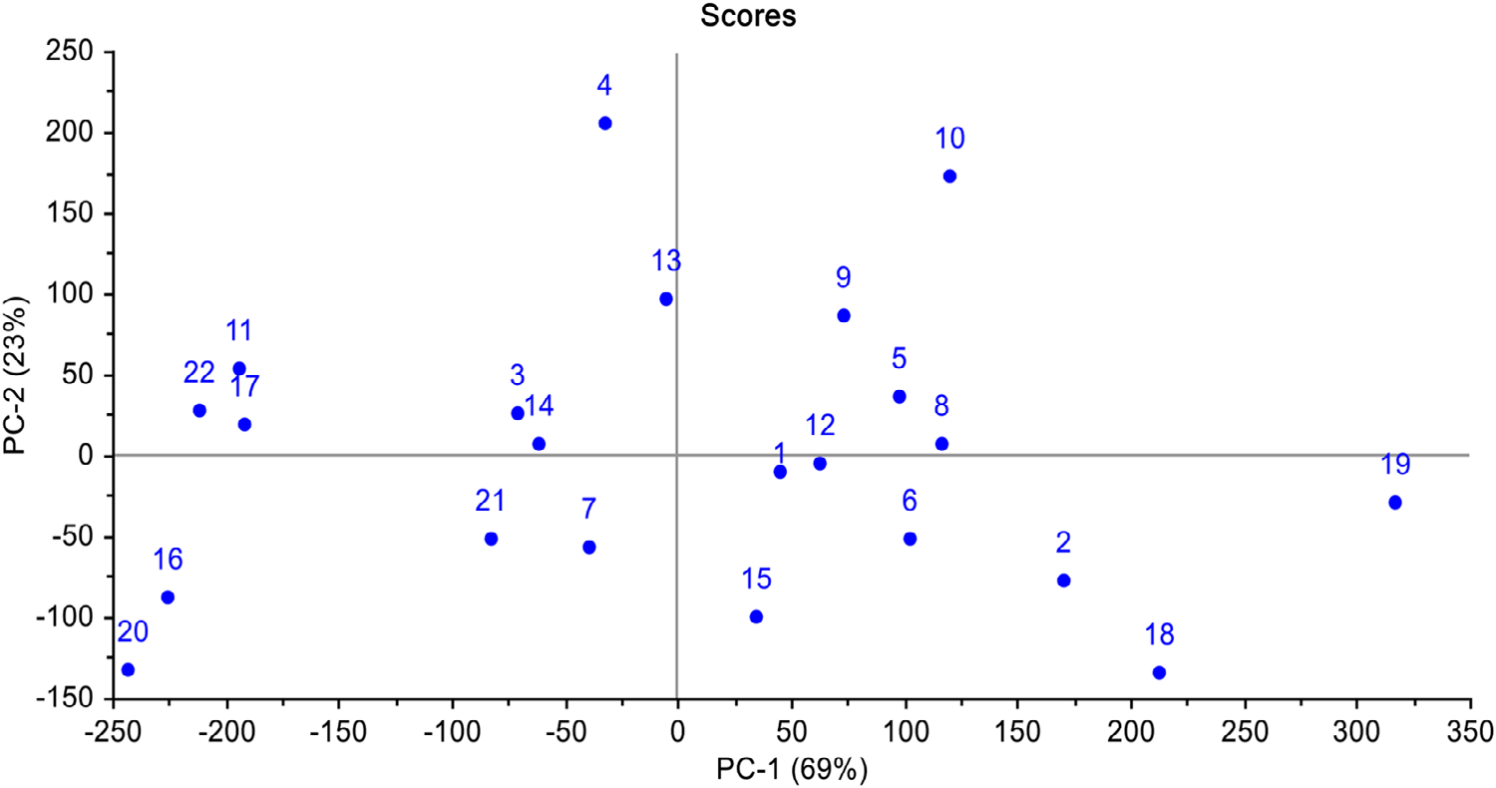
Score plot of 22 non‐wood samples (sample IDs and their raw material names are given in Table 1).

FT‐NIR spectroscopic data of non‐wood pulp samples are plotted in the score plot of principal component analysis (PCA) and shown in Figure 2. The plot indicates that the samples are scattered and distributed without following any pattern. It implies that there is no autocorrelation amongst the samples, and the samples are very suitable for developing any multivariate model [51]. Principal components (PCs) are newly constructed variables by transforming a large set of variables into a smaller one that still contains most of the information in the large set. They are constructed in such a way that the first PC (PC‐1) contains maximum variation amongst the data, then the second component (PC‐2) and so on.

Explained variance shows how much the newly constructed PCs have the ability to influence the target (dependent) variable. In this PCA, PC‐1 expresses 69% of the total variability and another 23% variability is expressed by PC‐2. Both can express 92% of the total variability amongst the data, and the results are shown in Table 2.

**TABLE 2:** Principal components and their contribution in explained variance.

	Explained Variance (%)						
Explained	PC‐1	PC‐2	PC‐3	PC‐4	PC‐5	PC‐6	PC‐7
Calibration	69.17	92.61	98.33	99.32	99.61	99.79	99.89
Validation	64.68	90.31	97.41	98.93	99.11	99.37	99.75

**FIGURE 3:**
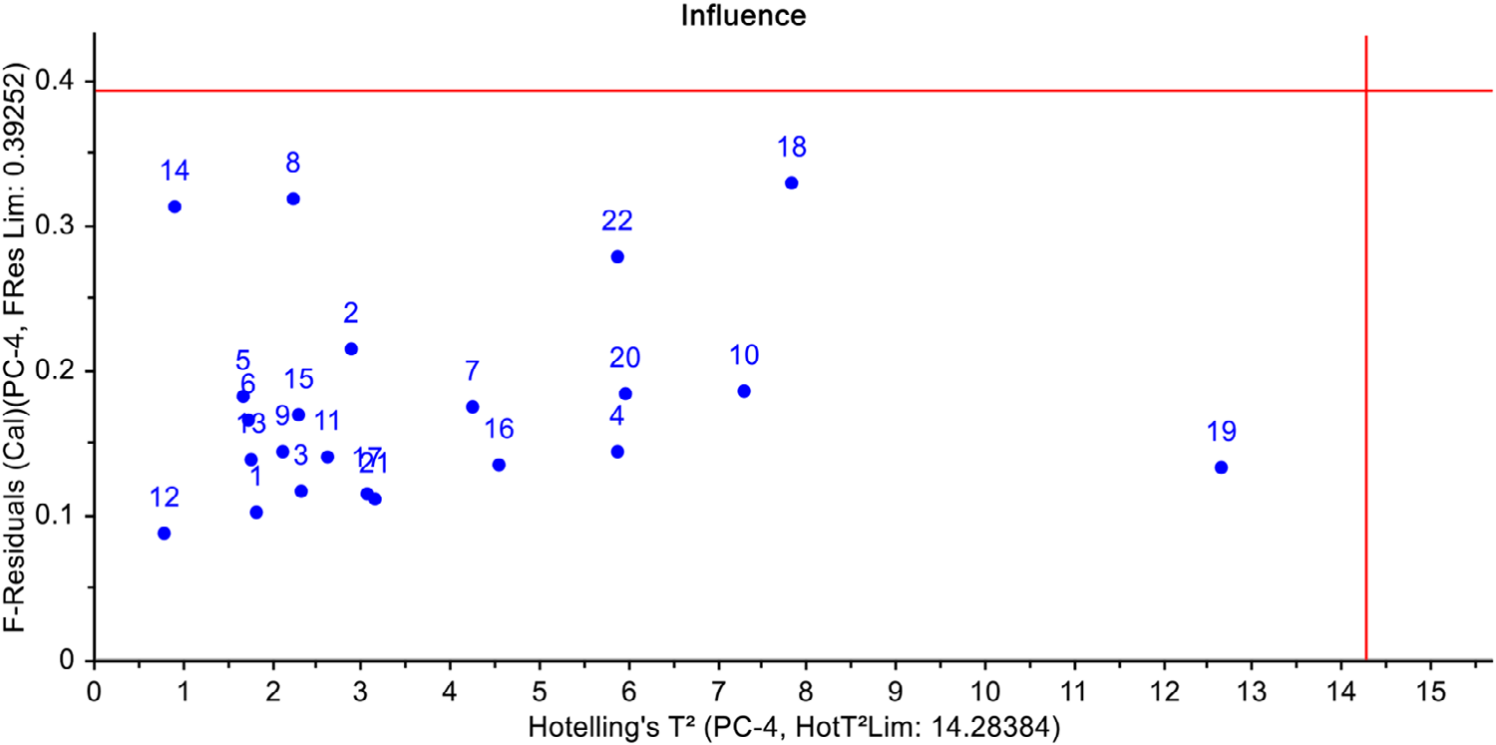
Influence Plot of PCA (sample IDs and their raw material names are given in Table 1).

In order to identify outlier (if any) in the sample, which might negatively influence the model efficiency, influence posts are used in the PCA. The influence plot of PCA shows the F‐residuals against Hotelling's *T* ^2^ (From the diagnostic analysis of the influence plot of PCA, it is clear that there is no outlier in the considered dataset. Therefore, multivariate calibration models could be developed whilst keeping all the samples in the analysis (Figure 3).

**FIGURE 4:**
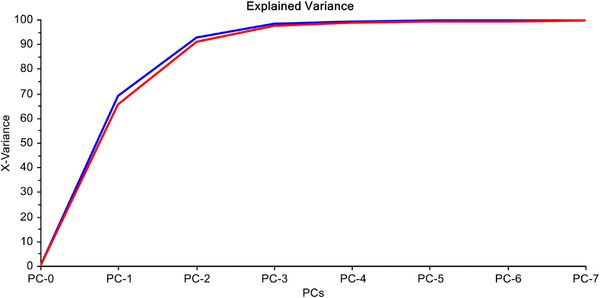
Explained variance plot.

First three PCs explain 98.33% of the total variation in the data. After that, remaining components have very insignificant contribution in expressing the variations in the data matrix (Figure 4).

**FIGURE 5:**
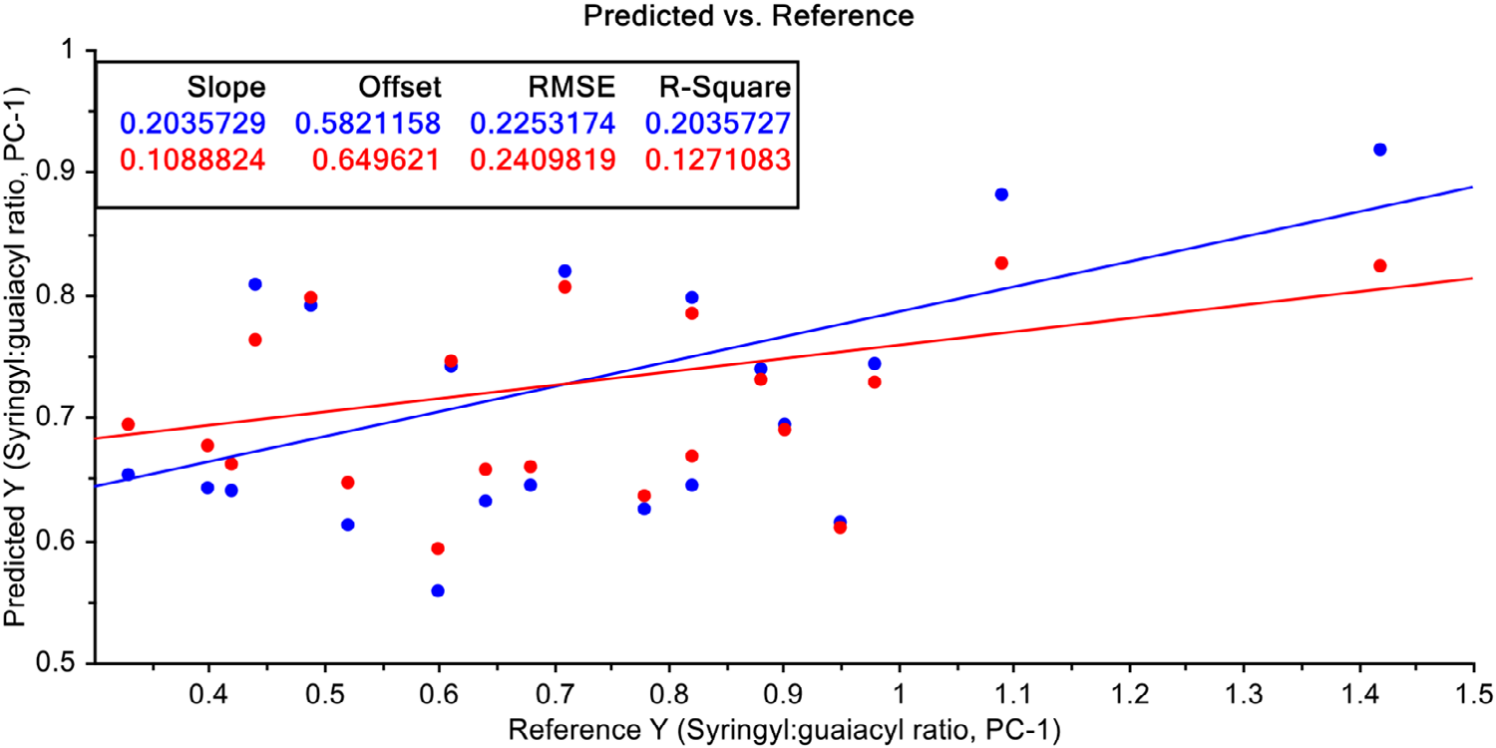
PCR model for syringyl/guaiacyl ratio quantification of raw FT‐NIR data.

PCR model for quantification of S/G ratio with raw FT‐NIR data of non‐wood pulps from agricultural residues is shown in Figure 5. Here, two model parameters namely, slope and offset are shown, and they are only 0.204 and 26.582 of the calibration dataset and 0.109 and 0.650 for sixfold CV datasets respectively. Moreover, two model efficiency parameters like root mean square error (RMSE) and *R* ^2^ are shown here, and they are 22.53% and 20.36% for calibration and 24.09% and 12.71% for validation data, respectively.

**FIGURE 6:**
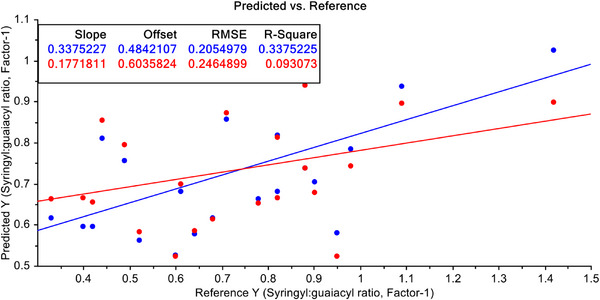
PLSR model for syringyl/guaiacyl ratio quantification of raw FT‐NIR data.

PLSR model for predicting S/G ratio content in the non‐wood pulp samples with raw FT‐NIT data is shown in Figure 6. Here, slope and offset are shown, and they are 0.338 and 0.484 of calibration dataset and 0.177 and 0.604 for CV datasets respectively. Moreover, the model efficiency parameters namely RMSE and *R* ^2^ are 20.55% and 33.75% for calibration and 24.65% and 9.31% for validation data, respectively. Here, neither the model parameters nor model efficiency parameters of the PLSR model with raw FT‐NIR data are satisfactory to use for predictive purposes. This poor performance of the model is due to the fact that the raw spectral data are taken from 22 different agricultural residue based non‐wood species.

**FIGURE 7:**
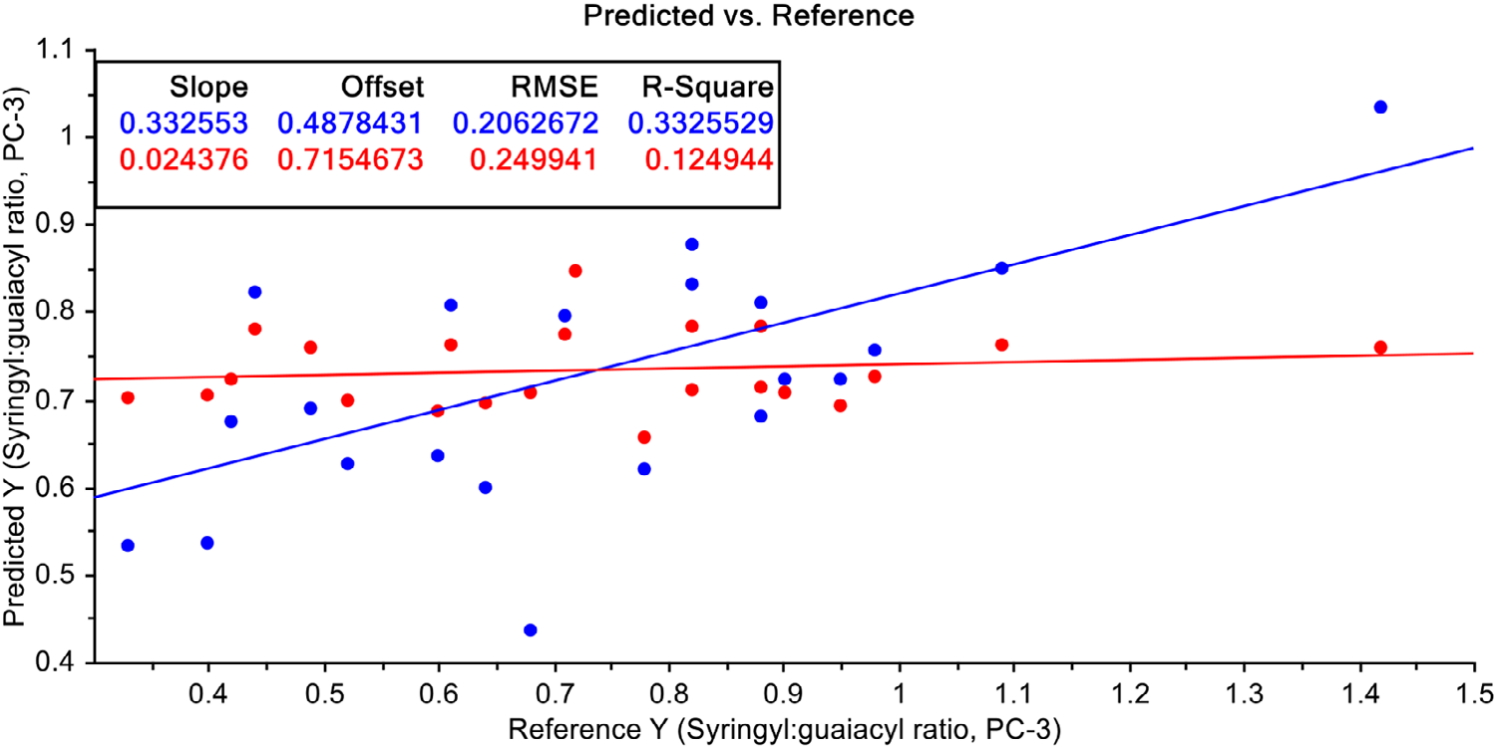
PCR model for syringyl/guaiacyl ratio quantification with pre‐treated data with Savitzky–Golay (S–G) filtering with 1st derivative of FT‐NIR data.

**FIGURE 8:**
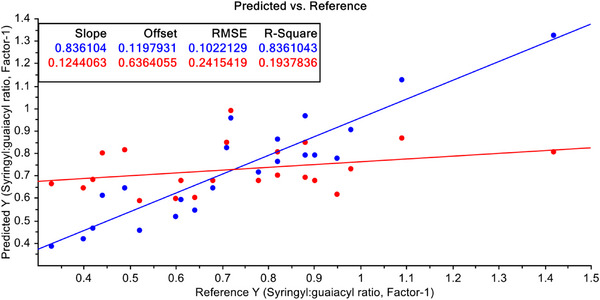
PLSR model for syringyl/guaiacyl ratio quantification with pre‐treated data with Savitzky–Golay (S–G) filtering with 1st derivative of FT‐NIR data.

In order to improve the predictability, the FT‐NIR data have been undergone some mathematical treatments. Firstly, the S–G filtering with 1st derivative of raw FT‐NIR data were performed. With these filtered data both PCR and PLSR models were developed again. PCR model with FT‐NIR data after S–G filtering with 1st derivative of raw data is shown in Figure 7. The model improved drastically after this treatment. Here, *R* ^2^ value has improved to 33.26% for calibration model and 12.49% for validation data. However, when the PLSR model with FT‐NIR data after S–G filtering with 1st derivative of raw data, the model improved dramatically (Figure 8), and it is an even better model than PCR both with calibration and validation dataset. Here, the *R* ^2^ values have improved to 83.61% for calibration model and 19.38% for validation data.

**FIGURE 9:**
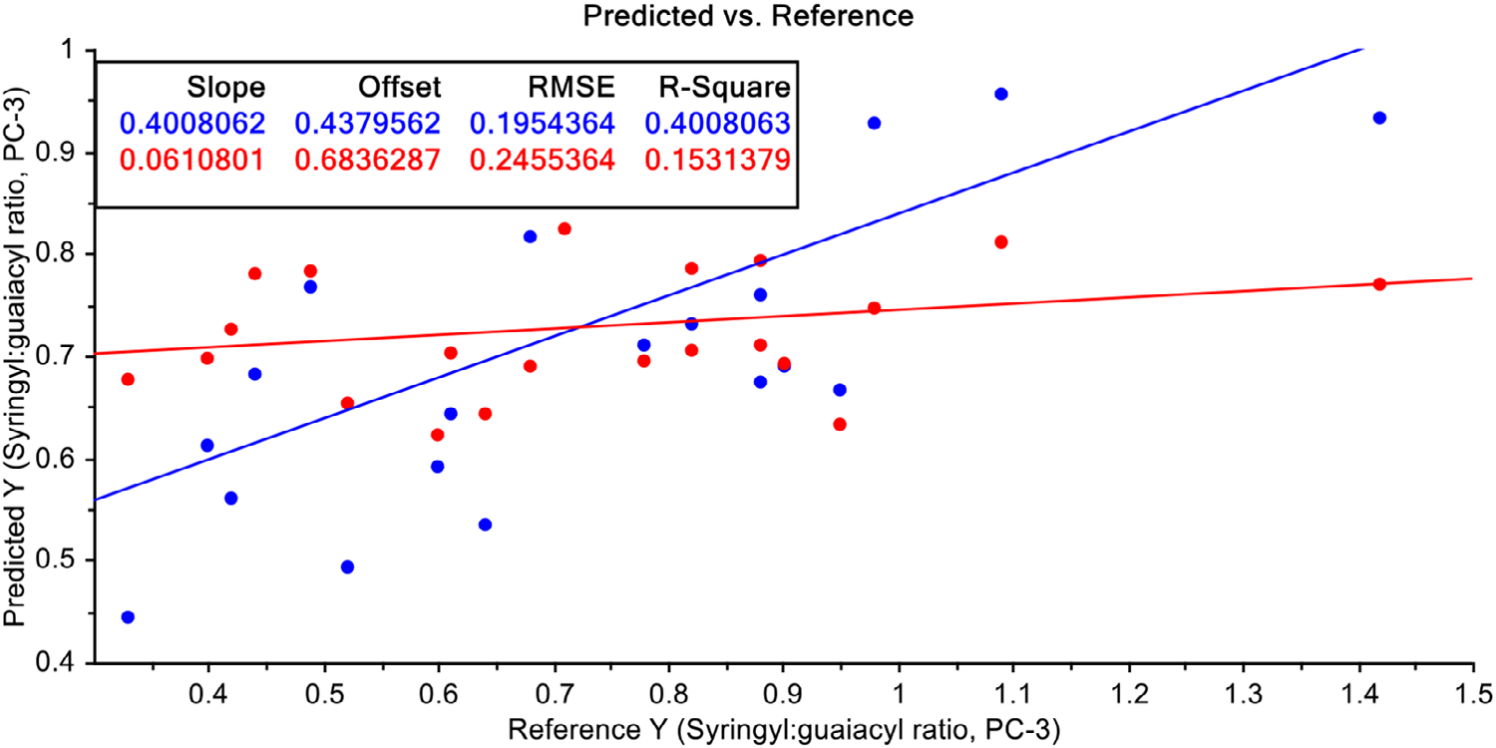
PCR model for syringyl/guaiacyl ratio quantification with pre‐treated data with Savitzky–Golay (S–G) filtering with 2nd derivative of FT‐NIR data.

**FIGURE 10:**
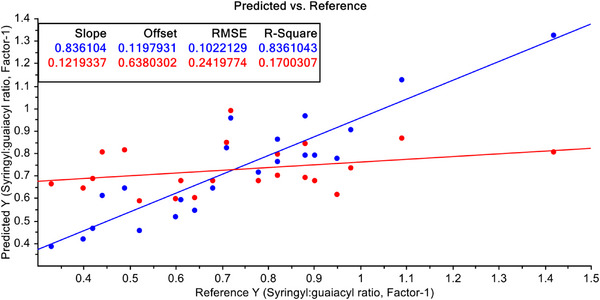
PLSR model for syringyl/guaiacyl ratio quantification with pre‐treated data with Savitzky–Golay (S–G) filtering with 2nd derivative of FT‐NIR data.

In quest of a better predictive model, the data have been treated again with S–G filtering with 2nd derivatives of FT‐NIR raw data next. With these treated data, PCR and PLSR model were developed again and models are presented in Figures 9 and 10. Performance of PCR model for prediction with pre‐treated data with S–G filtering with 2nd derivative of raw FT‐NIR data have is 40.08% for calibration and 15.31% for validation dataset. For prediction efficiency of PLSR model in terms of *R* ^2^ with S–G filtered data with 2nd derivative is very close to that of PLSR with S–G filtered data with 1st derivative for calibration dataset, and for validation data, the results are worse than with S–G filtered data with 1st derivative (Figure 10).

**FIGURE 11:**
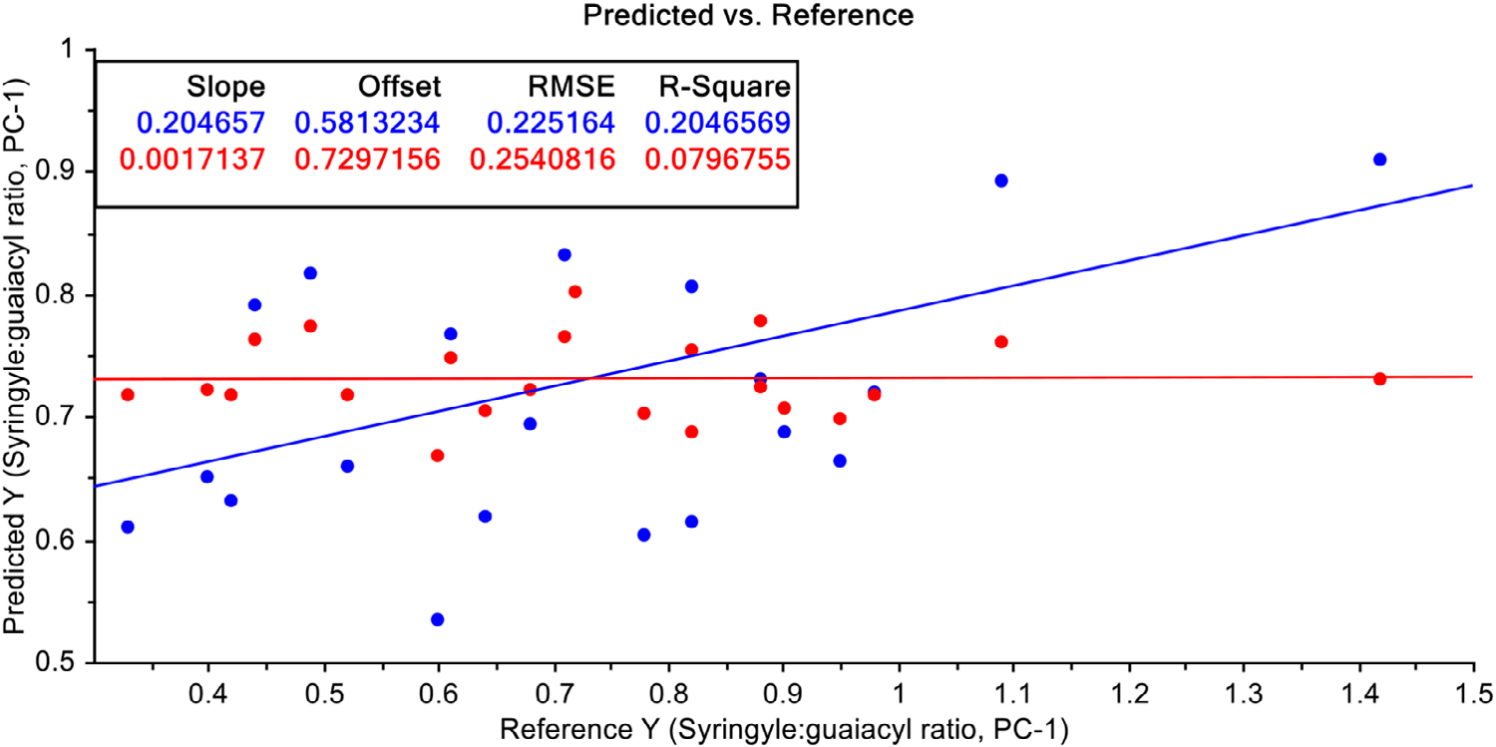
PCR model for syringyl/guaiacyl ratio quantification with pre‐treated data with Savitzky–Golay (S–G) filtering with 1st and 2nd derivative of FT‐NIR data.

**FIGURE 12:**
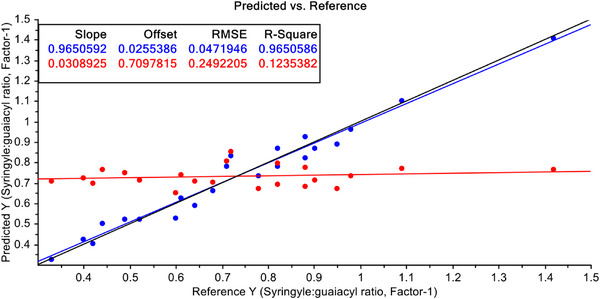
PLSR model for syringyl/guaiacyl ratio quantification with pre‐treated data with Savitzky–Golay (S–G) filtering with 1st and 2nd derivative of FT‐NIR data.

Further, to improve the predictive models, the data have been treated again with S–G filtering with 1st and 2nd derivatives of FT‐NIR raw data simultaneously. With these treated data PCR and PLSR model have been developed again and models are presented in Figures 11 and 12. PCR model with S–G filtering with 1st and 2nd derivatives of FT‐NIR raw data together would not be able to improve the predictive efficiency (Figure 11). A dramatic improvement has been noticed in case of PLSR model (*R* ^2^ = 96.51%) in case of calibration dataset. However, in case of validation data, *R* ^2^ value falls into 12.35% which indicates that the PLSR model becomes unstable, and this also could not be recommended for predictive purpose of S/G ratio in agricultural residue based non‐wood lignin.

**FIGURE 13:**
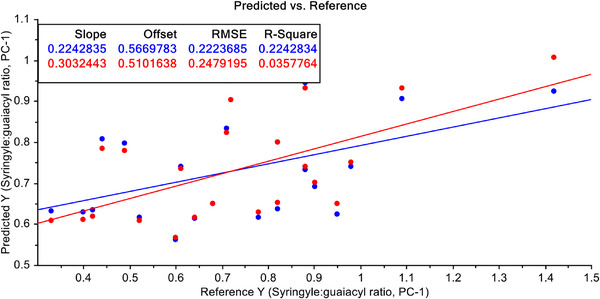
PCR model for syringyl/guaiacyl ratio quantification after leverage correction.

**FIGURE 14:**
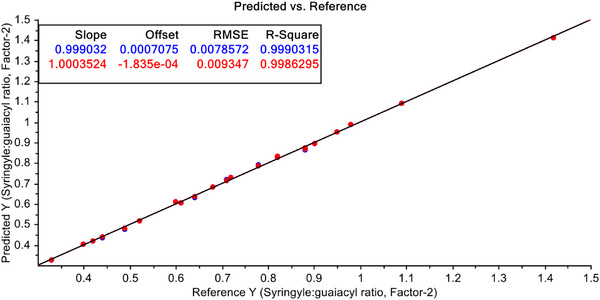
PLSR model for syringyl/guaiacyl ratio quantification after leverage correction.

Finally, Leverage correction has been used to improve the predictive efficiency of PCR and PLSR with pre‐treated FT‐NIR spectroscopic data with S–G filtered with 1st derivative. The results are shown in Figures 13 and 14, respectively. After pre‐treatment of FT‐NIR spectroscopic data with S–G filtering with 1st derivatives and leverage correction both for the PCR and PLSR models, the efficiency of the PCR model does not improve significantly (Figure 13), but a dramatic improvement of the PLSR model efficiency has been achieved, and *R* ^2^ value for calibration data is 99.90% and for validation dataset it is 99.86% (Figure 14). This indicates that this model is not only an efficient one for predicting S/G ratio in non‐wood lignin but also a very stable model for this purpose.

A comparative picture of predictive efficiencies of developed models in terms of *R* ^2^ values is shown in Table 3. Here the performance of the developed model with raw and treated FT‐NIR data both for calibration and validation dataset is presented. The best predictive performance is shown by FT‐NIR data when they are pre‐treated with S‐G filtering with 1st derivatives and leverage correction both for calibration (*R* ^2^ = 99.90%) and validation (*R* ^2^ = 99.86%) stages of modelling, even though the samples are from multispecies of non‐wood. The results obtained in the present work are much better than those from FT‐Raman‐PLSR, NIR‐PLSR and NIR‐PLS methods reported in the literature with uni‐species samples [26, 28, 40, 43, 44, 45].

**TABLE 3:** Coefficient of determination (*R* ^2^) of calibration models with raw and treated data.

Dataset	Raw Data		S–G Filtering with 1st Derivative		S–G Filtering with 2nd Derivative		S–G Filtering with 1st and 2nd Derivative		S–G Filtering with 1st Derivatives and Leverage Correction Data	
	PCR	PLSR	PCR	PLSR	PCR	PLSR	PCR	PLSR	PCR	PLSR
Calibration	20.35	33.75	33.26	83.61	40.08	83.61	20.47	96.51	22.42	99.90
Validation	12.71	9.31	12.49	19.38	15.31	17.00	7.97	12.35	3.58	99.86

## 4 Conclusions

Neither the PCR nor PLSR model shows satisfactory predictive performance with raw FT‐NIR data of non‐wood lignin from 22 different agricultural residues for quantification of S/G ratio, which is due to the variation amongst the spectral data from multispecies of samples. Therefore, some mathematical treatment is needed to pre‐process the data, that is, S–G filtering with 1st derivative and 2nd derivative individually and combined; indeed, the models have improved, but they are not still satisfactory for predictive use. In order to overcome this problem, leverage correction was used to improve the predictive efficiency of PCR and PLSR with pre‐treated FT‐NIR spectroscopic data with S–G filtered with 1st derivative. The PCR model did not improve significantly, but a dramatic improvement of PLSR model has been achieved. Now the PLSR model becomes an efficient and stable one for predicting S/G ratio in non‐wood lignin, and also a very stable model for this purpose.

Finally, the S/G ratio content in non‐wood lignin has been quantified by PLSR model with FT‐NIR data treated with S–G filtered with 1st derivative and leverage correction simultaneously with high efficiency both for calibration (*R* ^2^ = 99.90%) and validation (*R* ^2^ = 99.86%) dataset. Now, S/G ratio could be quantified in non‐wood samples only by using the FT‐NIR spectral data of the samples in PLSR model. Unlike other procedures, no further need of chemical use or laborious and time consuming steps to quantify S/G ratio in samples in a non‐destructive manner. This method could be applied to determine the amount of S/G ratio in lignin from similar sources, and this would be a non‐destructive, rapid and cost‐effective one.

## Author Contributions

**M. Nashir Uddin**: conceptualisation, writing – original draft. **Taslima Ferdous**: data curation, investigation. **Yangcan Jin**: investigation, methodology. **M. Mostafizur Rahman**: validation, visualisation. **M. Sarwar Jahan**: conceptualisation, supervision, writing – review and editing.

## Conflicts of Interest

The authors declare no conflicts of interest.

## Data Availability

Data sharing is not applicable to this article as no new data were created or analysed in this study.

## Appendix 1

### 1.1

(1) Bagasse (*Saccharum officinarum*), (2) bamboo (*Melocanna baccifera*), (3) banana (*Musa cavendish*) pseudo stem, (4) banana (*Musa cavendish*) leaf, (5) banana (*Musa cavendish*) peduncle, (6) cassava (*Manihot esculenta*) stalks, (7) chia stalk (*Salvia hispanica*), (8) corn stalk (*Zea mays*), (9) cotton stalk (*Gossypium arboreum L*), (10) dhaincha (*Sesbania aculeata*), (11) eggplant (*Solanum melongena*) stalks, (12) jute fibre (*Corchorus capsularis*), (13) jute stick (*Corchorus capsularis*), (14) kash (*Saccharum spontaneum*) stalks, (15) Kaun (*Seetaria‐ltalika*) straw, (16) mulberry stalk (*Morus* spp.), (17) mustard stalk (*Brassica juncea*), (18) okra stalk (*Abelmoschus esculentus*), (19) pineapple leaves (*Ananas comosus)*, (20) red lentil stalk (*Lens culinaris*), (21) rice straw (*Oryza sativa*) and (22) wheat straw (*Triticum monococcum* L.).

## References

[1] D. Piló‐Veloso , E. A. Nascimento , and S. A. L. Morais , “Isolamento E Análise Estrutural de Ligninas,” Quimica Nova 16, no. 5 (1993): 435–448.

[2] L. C. Barbosa , C. R. Maltha , V. L. Silva , and J. L. Colodette , “Determinação da Relação siringila/guaiacila da lignina em madeiras de eucalipto por pirólise acoplada à Cromatografia gasosa e Espectrometria de massas (PI CG/EM),” Química Nova 31 (2008): 2035–2041, 10.1590/S0100-40422008000800023.

[3] M. P. Cruz , L. C. Barbosa , C. R. Maltha , J. L. Gomide , and A. F. Milanez , “Chemical Characterization of Pitch in *Eucalyptus* Pulp and Paper Industry,” Química Nova 29 (2006): 459–466. [in Portuguese].

[4] C. F. Lima , L. C. A. Barbosa , C. R. Marcelo , F. O. Silvério , and J. L. Colodette , “Comparison Between Analytical Pyrolysis and Nitrobenzene Oxidation for Determination of Syringyl/Guaiacyl Ratio in *Eucalyptus* spp. Lignin,” BioResources 3, no. 3 (2008): 701–712.

[5] F. O. Silvério , L. C. Barbosa , C. R. Maltha , et al., “Effect of Storage Time on the Composition and Content of Wood Extractives in *Eucalyptus* Cultivated in Brazil,” Bioresource Technology 99, no. 11 (2008): 4878–4886. 17988861 10.1016/j.biortech.2007.09.066

[6] H. Yokoi , T. Nakase , Y. Ishida , et al., “Discriminative Analysis of *Eucalyptus camaldulensis* Grown From Seeds of Various Origins Based on Lignin Components Measured by Pyrolysis‐Gas Chromatography,” Journal of Analytical and Applied Pyrolysis 57, no. 1 (2001): 145–152.

[7] J. C. Del Río , A. Gutiérrez , M. Hernando , P. Landín , J. Romero , and A. T. Martínez , “Determining the Influence of Eucalypt Lignin Composition in Paper Pulp Yield Using Py‐GC/MS,” Journal of Analytical and Applied Pyrolysis 74 (2005): 104–109.

[8] F. J. González‐Vila , G. Almendros , J. C. Del Río , F. Martın , A. Gutiérrez , and J. Romero , “Ease of Delignification Assessment of Wood From Different *Eucalyptus* Species by Pyrolysis (TMAH)‐GC/MS and CP/MAS 13C‐NMR Spectrometry,” Journal of Analytical and Applied Pyrolysis 49, no. 1‐2 (1999): 295–305.

[9] S. K. Bose , R. C. Francis , M. Govender , T. Bush , and A. Spark , “Lignin Content Versus Syringyl to Guaiacyl Ratio Amongst Poplars,” Bioresource Technology 100, no. 4 (2009): 1628–1633, 10.1016/j.biortech.2008.08.046. 18954979

[10] M. Govender , T. Bush , A. Spark , S. K. Bose , and R. C. Francis , “An Accurate and Non‐Labor Intensive Method for the Determination of Syringyl to Guaiacyl Ratio in Lignin,” Bioresource Technology 100, no. 23 (2009): 5834–5839, 10.1016/j.biortech.2009.06.009. 19576762

[11] T. Ona , T. Sonoda , K. Ito , M. Shibata , T. Kato , and Y. Ootake , “Non‐Destructive Determination of Wood Constituents by Fourier Transform Raman Spectroscopy,” Journal of Wood Chemistry and Technology 17, no. 4 (1997): 399–417.

[12] G. Gellerstedt and J. Li , “An HPLC Method for the Quantitative Determination of Hexeneuronic Acid Groups in Chemical Pulps,” Carbohydrate Research 294 (1996): 41–51. 8962485 10.1016/s0008-6215(96)90615-1

[13] Y. Xie and S. Yasuda , “Difference of Condensed Lignin Structures in Eucalyptus Species,” Nordic Pulp & Paper Research Journal 19, no. 1 (2004): 18–21.

[14] F. Kačík , J. Ďurkovič , and D. Kačíková , “Structural Characterization of Lignin by Syringyl to Guaiacyl Ratio and Molecular Mass Determination,” in Lignin: Structural Analysis, Applications in Biomaterials and Ecological Significance (Nova Science Publishers, 2014), 67–89.

[15] C. A. Nunes , C. F. Lima , L. C. Barbosa , J. L. Colodette , A. F. G. Gouveia , and F. O. Silvério , “Determination of *Eucalyptus* spp Lignin S/G Ratio: A Comparison Between Methods,” Bioresource Technology 101, no. 11 (2010): 4056–4061, 10.1016/j.biortech.2010.01.012. 20133130

[16] J. Rodrigues , J. Graça , and H. Pereira , “Influence of Tree Eccentric Growth on Syringyl/Guaiacyl Ratio in *Eucalyptus globulus* Wood Lignin Assessed by Analytical Pyrolysis,” Journal of Analytical and Applied Pyrolysis 58 (2001): 481–489.

[17] J. Ralph and R. D. Hatfield , “Pyrolysis‐GC‐MS Characterization of Forage Materials,” Journal of Agricultural and Food Chemistry 39, no. 8 (1991): 1426–1437.

[18] F. O. Silverio , L. C. A. Barbosa , and D. Piló‐Veloso , “A Pirólise Como Técnica Analítica,” Quimica Nova 31 (2008): 1543–1552.

[19] J. C. del Río , A. Gutiérrez , I. M. Rodríguez , D. Ibarra , and Á. T. Martínez , “Composition of Non‐Woody Plant Lignins and Cinnamic Acids by Py‐GC/MS, Py/TMAH and FT‐IR,” Journal of Analytical and Applied Pyrolysis 79, no. 1‐2 (2007): 39–46.

[20] A. Nakagawa‐Izumi , Y. Y. H'ng , L. T. Mulyantara , R. Maryana , V. T. Do , and H. Ohi , “Characterization of Syringyl and Guaiacyl Lignins in Thermomechanical Pulp From Oil Palm Empty Fruit Bunch by Pyrolysis‐Gas Chromatography‐Mass Spectrometry Using Ion Intensity Calibration,” Industrial Crops and Products 95 (2017): 615–620.

[21] M. J. Rosado , J. Rencoret , G. Marques , A. Gutiérrez , and J. C. Del Río , “Structural Characteristics of the Guaiacyl‐Rich Lignins From Rice (*Oryza sativa* L.) Husks and Straw,” Frontiers in Plant Science 12 (2021): 640475. 33679856 10.3389/fpls.2021.640475 PMC7932998

[22] T. Ferdous , M. A. Quaiyyum , Y. Jin , M. S. Bashar , K. M. Yasin Arafat , and M. S. Jahan , “Pulping and Bleaching Potential of Banana Pseudo Stem, Banana Leaf and Banana Peduncle,” Biomass Conversion and Biorefinery 13 (2021): 893–904.

[23] T. Ferdous , M. A. Quaiyyum , A. Salam , and M. S. Jahan , “Pulping of Bagasse (*Saccrarum officinarum*), Kash (*Saccharum spontaneum*) and Corn Stalks (*Zea mays*),” Current Research in Green and Sustainable Chemistry 3 (2020): 100017.

[24] A. Alves , N. Gierlinger , M. Schwanninger , and J. Rodrigues , “Analytical Pyrolysis as a Direct Method to Determine the Lignin Content in Wood: Part 3. Evaluation of Species‐Specific and Tissue‐Specific Differences in Softwood Lignin Composition Using Principal Component Analysis,” Journal of Analytical and Applied Pyrolysis 85, no. 1‐2 (2009): 30–37.

[25] A. Alves , R. Simões , J. L. Lousada , J. Lima‐Brito , and J. Rodrigues , “Predicting the Lignin H/G Ratio of *Pinus sylvestris* L. Wood Samples by PLS‐R Models Based on Near‐Infrared Spectroscopy,” Holzforschung 74, no. 7 (2020): 655–662, 10.1515/hf-2019-0186.

[26] A. Alves , R. Simões , D. J. Stackpole , et al., “Determination of the Syringyl/Guaiacyl Ratio of *Eucalyptus globulus* Wood Lignin by Near Infrared‐Based Partial Least Squares Regression Models Using Analytical Pyrolysis as the Reference Method,” Journal of Near Infrared Spectroscopy 19, no. 5 (2011): 343–348, 10.1255/jnirs.946.

[27] A. Biancolillo and F. Marini , “Chemometric Methods for Spectroscopy‐Based Pharmaceutical Analysis,” Frontiers in Chemistry 6 (2018): 576, 10.3389/fchem.2018.00576. 30519559 PMC6258797

[28] C. P. Diniz , D. Grattapaglia , S. D. Mansfield , and L. F. de Alencar Figueiredo , “Near‐Infrared‐Based Models for Lignin Syringyl/Guaiacyl Ratio of *Eucalyptus benthamii* and *E. pellita* Using a Streamlined Thioacidolysis Procedure as the Reference Method,” Wood Science and Technology 53, no. 3 (2019): 521–533, 10.1007/s00226-019-01090-3.

[29] A. Alves , A. Santos , D. da Silva Perez , et al., “NIR PLSR Model Selection for Kappa Number Prediction of Maritime Pine Kraft Pulps,” Wood Science and Technology 41 (2007): 491–499, 10.1007/s00226-007-0130-0.

[30] V. Kothiyal , Jaideep , S. Bhandari , H. S. Ginwal , and S. Gupta , “Multi‐Species NIR Calibration for Estimating Holocellulose in Plantation Timber,” Wood Science and Technology 49 (2015): 769–793, 10.1007/s00226-015-0720-1.

[31] R. Meder , S. Gallagher , K. L. Mackie , H. Böhler , and R. R. Meglen , “Rapid Determination of the Chemical Composition and Density of *Pinus radiata* by PLS Modelling of Transmission and Diffuse Reflectance FTIR Spectra,” Holzforschung 53 (1999): 261–266.

[32] M. N. Uddin , J. Nayeem , M. S. Islam , and M. S. Jahan , “Rapid Determination Method of Dissolving Pulp Properties by Spectroscopic Data and Chemometrics,” Biomass Conversion and Biorefinery 9 (2019): 585–592, 10.1007/s13399-019-00383-8.

[33] M. N. Uddin , T. Ferdous , Z. Islam , M. S. Jahan , and M. A. Quaiyyum , “Development of Chemometric Model for Characterization of Non‐Wood by FT‐NIR Data,” Journal of Bioresources and Bioproducts 5, no. 3 (2020): 196–203, 10.1016/j.jobab.2020.07.005.

[34] O. T. Okan , I. Deniz , and S. Tiryaki , “Application of Artificial Neural Networks for Predicting Tensile Index and Brightness in Bleaching Pulp,” Maderas Ciencia y Tecnología 17, no. 3 (2015): 571–584, 10.4067/S0718-221X2015005000051.

[35] A. Santos , O. Anjos , and H. Pereira , “Estimation of *Acacia melanoxylon* Unbleached Kraft Pulp Brightness by NIR Spectroscopy,” Forest Systems 24, no. 2 (2015): eRC03, 10.5424/fs/2015242-07580.

[36] M. N. Uddin , S. Ahmed , S. K. Ray , M. S. Islam , A. H. Quadery , and M. S. Jahan , “Method for Predicting Lignocellulose Components in Jute by Transformed FT‐NIR Spectroscopic Data and Chemometrics,” Nordic Pulp & Paper Research Journal 34, no. 1 (2019): 1–9, 10.1515/npprj-2018-0018.

[37] T. F. Yeh , T. Yamada , E. Capanema , H. M. Chang , V. Chiang , and J. F. Kadla , “Rapid Screening of Wood Chemical Component Variations Using Transmittance Near‐Infrared Spectroscopy,” Journal of Agricultural and Food Chemistry 53, no. 9 (2005): 3328–3332, 10.1021/jf0480647. 15853367

[38] M. N. Uddin , S. K. Ray , M. S. Islam , J. Nayeem , and M. S. Jahan , “Development of Method for Rapid Prediction of Chemical Components of Dhaincha Using FT‐NIR Spectroscopy and Chemometrics,” Journal of Science and Technology for Forest Products and Processes 6, no. 4 (2017): 22–28.

[39] L. Liang , T. Wu , G. Fang , et al., “Predicting Bleachability of *Eucalyptus* Mechanical Pulp by Moisture Content‐Dependent Near‐Infrared Spectroscopy,” Industrial Crops and Products 180 (2022): 114730, 10.1016/j.indcrop.2022.114730.

[40] P. Ramadevi , D. V. Hegde , M. Varghese , R. Kamalakannan , S. P. Ganapathy , and D. S. Gurumurthy , “Evaluation of Lignin Syringyl/Guaiacyl Ratio in *Eucalyptus camaldulensis* Across Three Diverse Sites Based on Near Infrared Spectroscopic Calibration Modelling With Five *Eucalyptus* Species and Its Impact on Kraft Pulp Yield,” Journal of Near Infrared Spectroscopy 24, no. 6 (2016): 529–536, 10.1255/jnirs.1251.

[41] U. P. Agarwal , S. A. Ralph , D. Padmakshan , S. Liu , and C. E. Foster , “Estimation of Syringyl Units in Wood Lignins by FT‐Raman Spectroscopy,” Journal of Agricultural and Food Chemistry 67, no. 15 (2019): 4367–4374. 30916944 10.1021/acs.jafc.8b06707

[42] L. Sun , P. Varanasi , F. Yang , D. Loqué , B. A. Simmons , and S. Singh , “Rapid Determination of Syringyl: Guaiacyl Ratios Using FT‐Raman Spectroscopy,” Biotechnology and Bioengineering 109, no. 3 (2012): 647–656, 10.1002/bit.24348. 22012706

[43] T. Ona , T. Sonoda , K. Ito , et al., “Non‐Destructive Determination of Lignin Syringyl/Guaiacyl Monomeric Composition in Native Wood by Fourier Transform Raman Spectroscopy,” Journal of Wood Chemistry and Technology 18, no. 1 (1998): 43–51.

[44] J. S. Lupoi , S. Singh , M. Davis , et al., “High‐Throughput Prediction of Eucalypt Lignin Syringyl/Guaiacyl Content Using Multivariate Analysis: A Comparison Between Mid‐Infrared, Near‐Infrared, and Raman Spectroscopies for Model Development,” Biotechnology for Biofuels 7 (2014): 1–14, 10.1186/1754-6834-7-93. 24955114 PMC4064109

[45] R. Prado , L. Weigand , S. M. Zahari , et al., “An Easy and Reliable Method for Syringyl: Guaiacyl Ratio Measurement,” Tappi Journal 16, no. 3 (2017): 145–152.

[46] M. Zeaiter , J. M. Roger , and V. Bellon‐Maurel , “Robustness of Models Developed by Multivariate Calibration. Part II: The Influence of Pre‐Processing Methods,” TrAC Trends in Analytical Chemistry 24, no. 5 (2005): 437–445, 10.1016/j.trac.2004.11.023.

[47] T. Naes , T. Isaksson , T. Fearn , and T. Davies , A User Friendly Guide to Multivariate Calibration and Classification (NIR Publications, 2002).

[48] H. Martens and T. Naest , Multivariate Calibration (John Wiley & Sons Ltd., 1989).

[49] B. S. Everitt , Cambridge Dictionary of Statistics (Cambridge University Press, 2002).

[50] T. Næs and M. R. Ellekjær , “Cross‐Validation and Leverage‐Correction Revisited,” NIR News 4, no. 1 (1993): 8–9.

[51] I. T. Jolliffe , Principal Component Analysis, 2nd ed. (Springer, 2002).

